# A near real-time web-system for predicting fire spread across the Cerrado biome

**DOI:** 10.1038/s41598-023-30560-9

**Published:** 2023-03-24

**Authors:** Ubirajara Oliveira, Britaldo Soares-Filho, Hermann Rodrigues, Danilo Figueira, Leticia Gomes, William Leles, Christian Berlinck, Fabiano Morelli, Mercedes Bustamante, Jean Ometto, Heloísa Miranda

**Affiliations:** 1 Center for Remote Sensing, Federal University of Minas Gerais, Belo Horizonte, Brazil; 2 Federal University of Brasília, Brasília, Brazil; 3 Chico Mendes Institute for the Conservation of Biodiversity, Brasília, Brazil; 4 The National Institute for Space Research, São Paulo, Brazil

**Keywords:** Environmental impact, Fire ecology

## Abstract

Wildfires are aggravating due to climate change. Public policies need territorial intelligence to prevent and promptly fight fires, especially in vast regions like Brazil. To this end, we have developed a fire-spread prediction system for the Brazilian Cerrado, the biome most affected by wildfires in South America. The system automatically uploads hot pixels and satellite data to calculate maps of fuels loads, vegetation moisture, and probability of burning for simulating fire spread thrice a day for the entire Cerrado at 25 ha and for nine conservation units at 0.04 ha spatial resolution. In both versions, the model attains 65–89% of spatial match. Model results together with ancillary data, e.g., historical burned areas and annual CO_2_ emissions from fires, are available on an interactive web-platform that serves as a tool for fire prevention and fight, particularly in the selected conservation units where the platform is being used for daily operations.

## Introduction

High-impact fires are expanding in Brazil^1^. Over the last two decades, those fires, characterized by extensive burned areas, frequent recurrence, high burning intensity and predominance in the dry season, have intensified, especially in the Brazilian Cerrado, by far the most affected biome in South America^2^. And this trend is likely to exacerbate due to climate and land-use change^1^. Wildfires highly impair the native vegetation^1^ and the biodiversity it shelters, entailing greenhouse gas emissions^3^, losses of ecosystem services^4–6^, and also affecting regional economies^7^ and human health^8,9^. For example, high-impact fires between 2000 and 2019 imposed a 19% reduction in the photosynthetic activity of fire-affected plants in the Brazilian Cerrado^1^, together with an annual drop of 5% in its biomass stocks^3^, despite the fire-resilience ecology of this biome^10,11^.

Unsurprisingly, firefighting has become increasingly difficult and costly all over the world^12^. In response, new tools, including online platforms, are emerging to help prevent and combat wildfires. Some global initiatives, such as the FIRMS^2^, provide satellite data on hot pixels (signal detection of radiance from fire flames at ± 1000 K)^13^, along with historical data on burned areas. Other regional initiatives put together high spatial resolution satellite imagery with climate and topographic data to generate fire risk maps, e.g.: Digital Earth Australia Hotspots (DISARM)^14^; European Forest Fire Information System^15^; Fire Information for Resource Management System US/Canada^16^; Amazon Dashboard^17^; INPE-Queimadas^18^; Ontario Forest Fire Info Map^19^; Wildland Fire Decision Support System^20^, and Wildfire Risk to Communities^21^. At the same time, market solutions for fighting fires are proliferating with private companies selling online services, including fire alerts based, for example, upon smoke plume detection^22–26^.

Although the science of fire behavior has made great strides since the 1940s^27^, thus far most of the aforementioned systems basically provide maps of fire risk based on environmental and climatic conditions of a given time, which in general have a medium predictive capability^28^. Therefore, there remains a need for near real-time systems capable of predicting the dynamics of fire behavior and thereby its propagation across vast regions as a function of terrain, vegetation structure and moisture, and fuel loads.

The science of fire behavior has a long tradition. Several models, such as FARSITE^29^, Prometheus^30^, and Spark^31^, have been developed in order to help combat, prevent and manage forest fires^27,32–34^. Those models hold a variety of approaches, encompassing from extremely simple representations, such as those of empirical models^35^ based on cellular automata framework^36–39^, to very complex ones that represent the interactions between the atmosphere and biosphere^40–42^. To illustrate some examples, the FARSITE model employs the Huygens principle to simulate the behavior of fire using equations of ellipses by assuming that a burning will have an ellipsoidal shape determined by predictor variables, i.e., fuel loads, wind, moisture and vegetation structure, temperature and topography^29^. The Prometheus model uses a cellular automaton to simulate fire spread based on predictor variables similar to those of FARSITE^30^. In turn, the Spark model generates a fire probability surface using Monte Carlo simulation, also employing variables equivalent to those of the former models^31^.

Despite the wide availability of fire spread models alongside the relative success of some in predicting fire behavior, such as FARSITE^43^, their low usability can be an obstacle and even turn them impractical during emergency situations^44^, since they require lots of spatial data–e.g., topography, land-use, wind, vegetation structure and moisture, temperature and fuel loads– that must be obtained, processed and input by the user for running the simulations, what is usually time-demanding and needs expertise.

Currently, advancements in the science of fire, together with the readiness of remote sensed imagery and climate data in both high spatial and temporal resolutions^45^, have allowed to model fire behavior specifically for the various environmental conditions of the terrestrial biomes. In addition, enhanced computer power at a low cost and the free availability of high-performance and user-friendly environmental modeling platforms^47^, alongside advances in automated data acquisition and processing^3,48^, have made it possible to develop near real-time simulations of fire propagation across large geographic areas at a fine spatial scale^49^.

Simulation results in the form of charts and maps available online and accessible to a broad range of users can thus be an effective means to help prevent and fight wildfires (e.g., PyreCast and FireSim^50,51^). The underlying idea is that a user-friendly multimedia platform would allow access to a wide public who necessarily do not hold technical skills, so they could quickly interpret the visual results it portrays. With this in mind, we have developed a fully automated online platform (https://csr.ufmg.br/fipcerrado/en/), which processes remote sensed imagery (MODIS and Sentinel-2) together with climate and terrain data to run near real-time (thrice a day) simulations of fire spread across the entire Brazilian Cerrado at 25 ha spatial resolution (500-m pixel) and at a fine spatial resolution of 0.04 ha (20-m pixel) for the encompassing regions of nine conservation units in this biome. Named as FISC-Cerrado (Fire, Ignition, Spread and Carbon Cycling), the system was developed under the auspices of the Forest Investment Program (FIP-Cerrado), a joint initiative by the Federal University of Minas Gerais (UFMG) and the National Space Research Institute (INPE) with collaboration of other research institutions, and sponsored by the World Bank Project no. P143185. Here, we report the underlying development, innovations, operation, and utilization of this system.

## The FISC-Cerrado system

**Figure 1:**
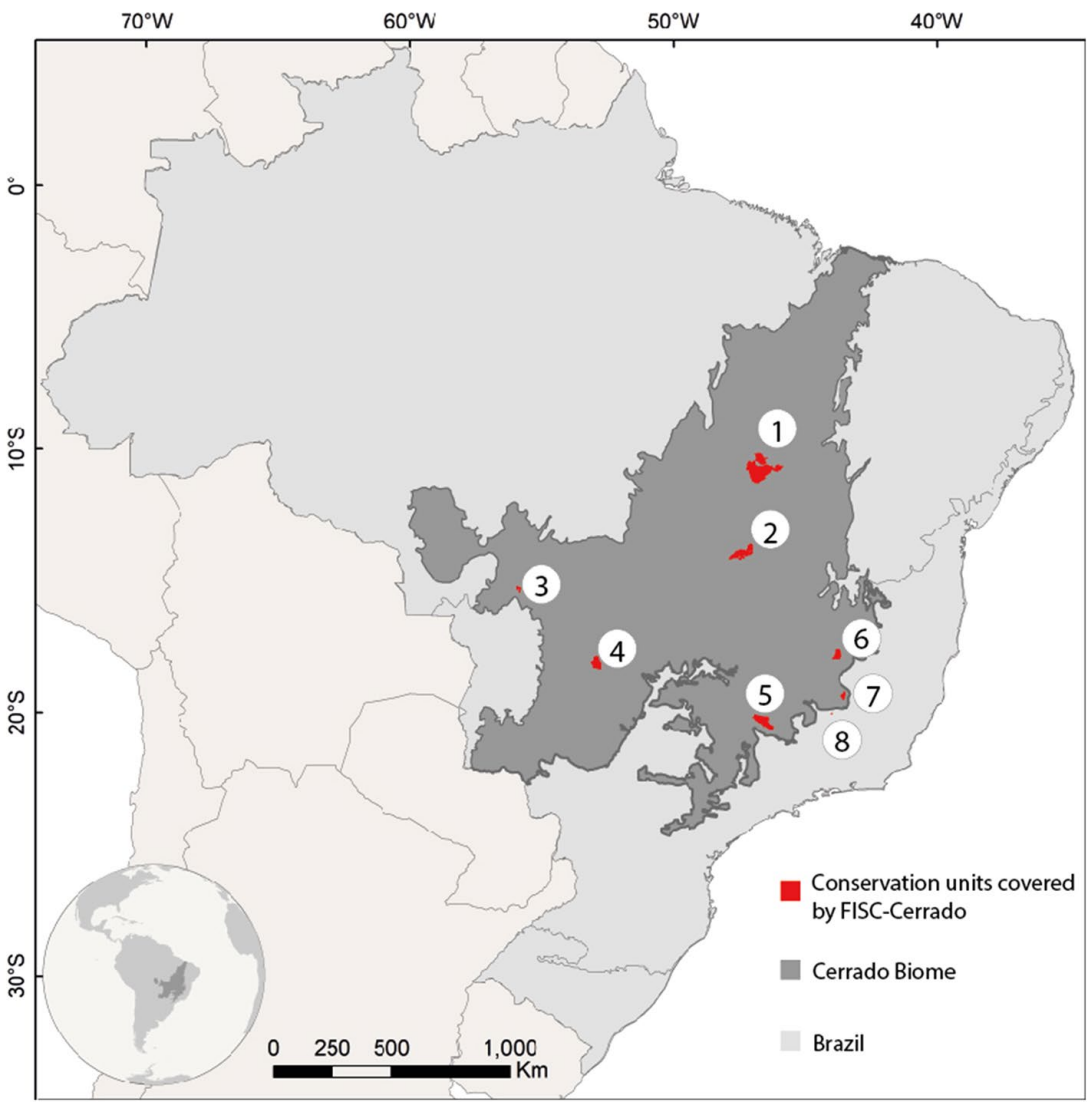
Cerrado biome and regions of the nine conservation units covered by FISC-Cerrado. 1– Jalapão State Park and Serra Geral do Tocantins Ecological Station; 2—Chapada dos Veadeiros National Park; 3—Chapada dos Guimarães National Park; 4—Emas National Park; 5—Serra da Canastra National Park; 6—Sempre-Vivas National Park; 7—Serra do Cipó National Park; 8—Serra do Rola Moça State Park. Map created in ArcGIS 10.1 (http://www.esri.com).

The FISC system runs automatically, thrice a day, at 6 am, 10 am and 3 pm for the Cerrado as a whole and for the regions of seven national conservation units (CUs), under the management of the Chico Mendes Institute for Biodiversity Conservation (ICMbio), and two state parks. These specific hours were selected in accordance with the daily work schedule of CUs' brigades. Those CUs are: (1) Chapada dos Veadeiros National Park, (2) Serra do Cipó National Park, (3) Serra da Canastra National Park, (4) Emas National Park, (5) Chapada dos Guimarães National Park, (6) Jalapão State Park, (7) Sempre-Vivas National Park, (8) Serra do Rola Moça State Park and (9) Serra Geral do Tocantins Ecological Station (Fig. 1).

**Figure 2:**
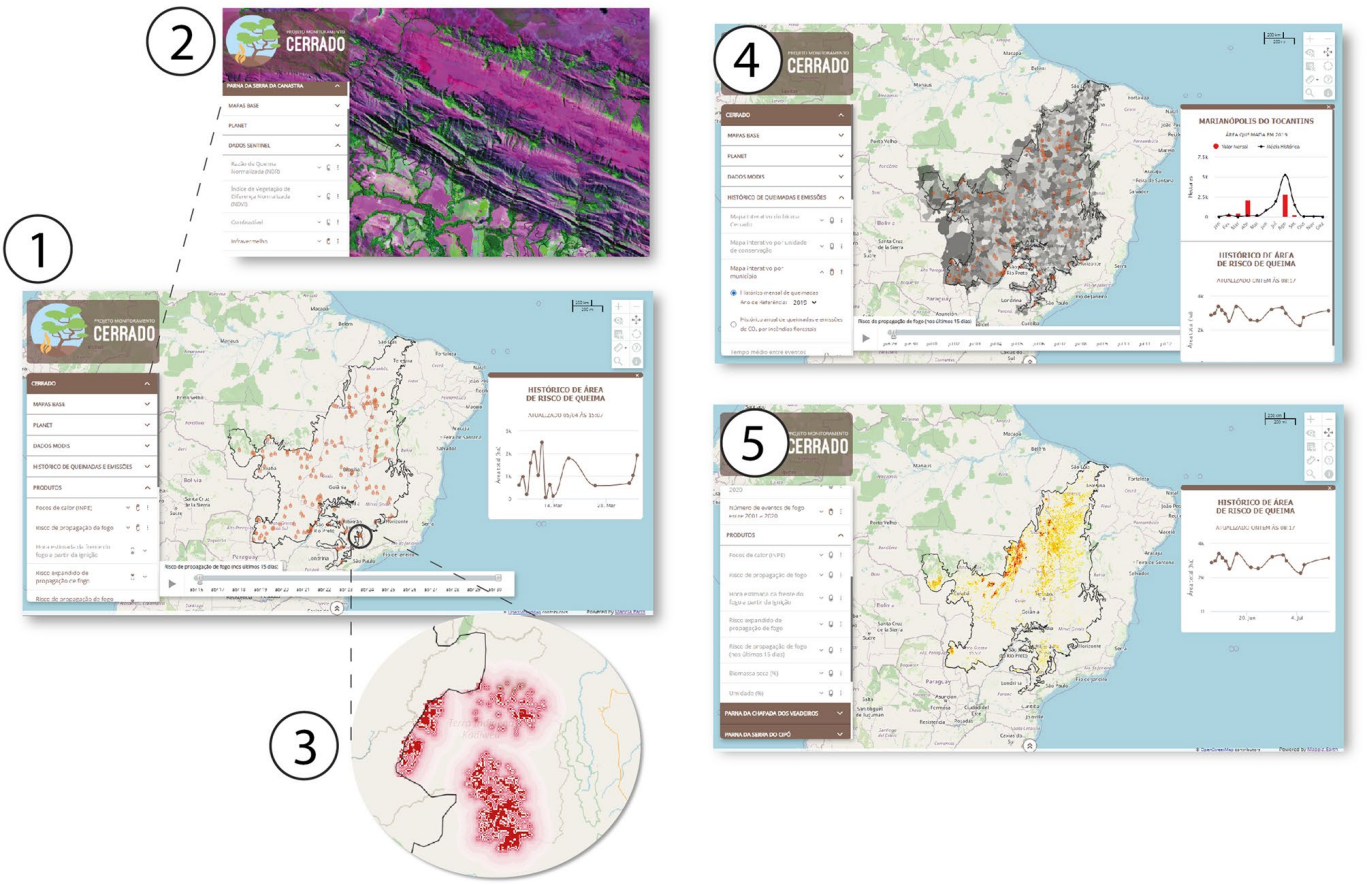
FISC-Cerrado web interface. 1—home page depicting hot pixels; 2—high spatial resolution map of fuels loads; 3—zoom on the map of fire spread risk; 4—graphs from map query showing the frequency of fires for the year of reference (bars) and its historical average per municipality; 5—frequency of burned areas. Maps created using Dinamica EGO (https://dinamicaego.com/).

The system steps consist of (1) download of hot pixels from ten satellites made available by INPE-Queimadas^52^ (Table S1, Online Supplementary Materials), MODIS^46^ and Sentinel-2 images^45^, and wind maps from the NOAA/NCEP Global Forecast System^53^; (2) image processing to generate maps of fuel loads^3^, vegetation moisture^54^ and probability of burning^3^ that are inputs for simulating fire spread; (3) execution of the fire spread model for the Cerrado and nine of its CUs thrice a day (simulations thus forecast fire spread for the next 8 h); (4) post-processing of resulting maps for the Cerrado and for the CUs along with tabular data for display and download at a web-map server platform. In addition to maps of fire spread, the platform brings together a series of ancillary data which are constantly updated, such as hot pixels from Queimadas/INPE^52^, maps of fuel loads and vegetation moisture^54^, CO_2_ emissions from fires^3^, historical records of burned areas over the last two decades per biome, municipality and conservation units, including their number of events, extent, recurrence, mean time interval between events and resulting fire intensity^1,55^. Along with cartographic and chart visualization and querying, all outputs (maps and tables) are publicly available for download in format of csv, geotiff and kmz files, the latter for visualization on Google Earth (Fig. 2).

The model builds upon our previous experience in simulating in a spatially explicit way forest fires in the Amazon^56,57^. Nevertheless, FISC-Cerrado brings up a brand-new design aimed to closely represent the fire ecology in the Cerrado as well as to improve the propagation mechanism based on empirical experiments^35,55,58^. All model components are developed as submodels and stored as new operators within the fire library-tab of Dinamica EGO freeware (www.dinamicaego.com). Dinamica EGO version 7.* takes advantage of full parallel processing^59^. Its execution system uses a variable number of execution threads (called workers) boosted by task-stealing algorithms to provide load balancing and flexibility for running simultaneous tasks. In theory, all model components can run in parallel, including independent operators, loops, and map tiles^60^. This architecture reduces drastically the execution time of complex models that run either locally or on the cloud. For building the online platform, we employ our in-house map server (https://mappia.earth/). Mappia freeware offers a set of customizable ready-to-use tools called elements that are assembled to develop map server platforms with various designs and layouts. Mappia elements allow a wide set of user interactions, such as inspect values, apply map algebra, display time-series maps and create interactive charts. Furthermore, Mappia can integrate multiple data sources from online databases, like the Planet imagery, also available on the FISC-Cerrado platform (Fig. 2).

## Model inputs and setup

**Figure 3:**
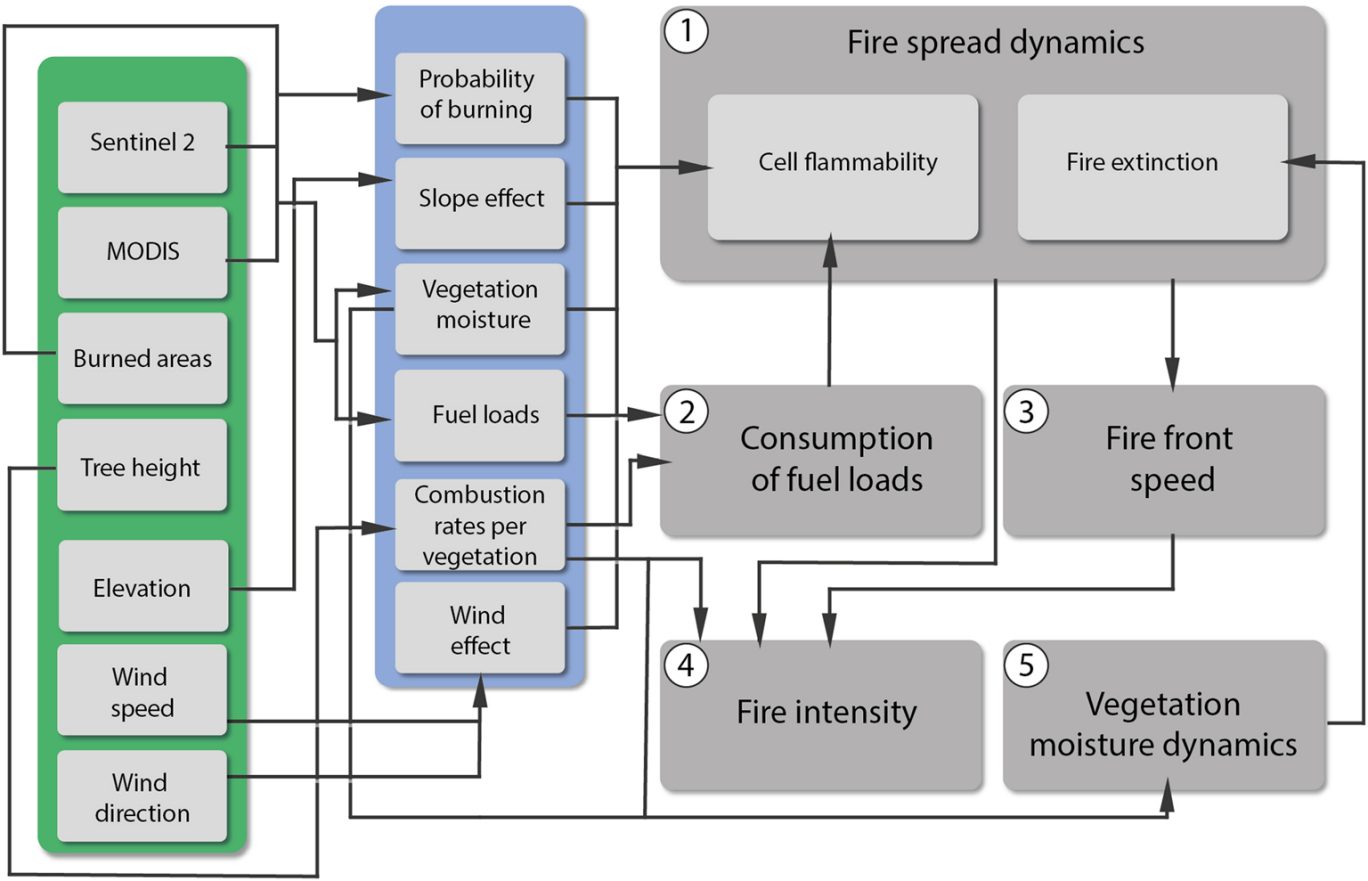
FISC-Cerrado main components. Input data (green), preprocessing of input data (blue) for feeding the cellular automata modules (gray).

The fire propagation model uses as inputs: (1) hot pixel data; (2) a map of probability of burning given the availability of fuel loads and historical records of burned areas^3^; (3) a map of dry biomass percentage used as fuel loads^3^; (4) the Normalized Difference Water Index (NDWI) as a proxy for vegetation moisture; (5) a digital elevation map; (6) maps of wind speed and direction, and (7) combustion rates per vegetation type^61^, and (8) a series of numeric constants from fire behavior experiments (Fig. 3, Table S2).

### Ignition sources

As ignition sources for triggering the propagation of a fire, the model employs the hot pixels from INPE-Queimadas, which collects and makes available hot pixel data from the satellites Aqua, Terra, GOES-16, NOAA-18, NOAA19, MSG-03, METOP-B, METOP-C, and NPP-375 (Table S1). These data are downloaded and filtered per every time interval between model runs.

### Satellite imagery

We chose to employ data that could be updated and processed automatically. In this way, the platform downloads MODIS images^62^, on a daily basis, and Sentinel-2^63^, whenever new images are available (usually between three and five days). For the Cerrado as whole, the model employs MODIS images, bands 1, 2, 3, 4, 6, and 7 as well as the burned area product^62,64^ at 500 m spatial resolution. For the selected CUs, the model uses Sentinel-2, spectral bands 2, 3, 4, 8, 11 and 12 at 20 m spatial resolution^63^. Both sets of images are assembled to form continuous mosaics and then processed to generate the variables used as input for modeling fire behavior and propagation, namely: a vegetation moisture index, percentage of dry biomass (fuel loads) and the probability of burning.

### Wind speed and direction

The model obtains every three hours wind data from the collection of Atmospheric Models of the NOAA/NCEP Global Forecasting System^53^*.* Their spatial resolution is 0.5° (≈ 50 km), and the wind is calculated at 10 m from the Earth's surface.

### Elevation

Shuttle Radar Topography Mission (SRTM) data—spatial resolution of 30 m—^66^ are used to derive the up-and-down slopes from a fire front.

### Fuel loads consumption rate per vegetation type

Estimate of combustion rates is key for modeling fuel loads dynamics during fire events as well as to predict the extinguishment of a fire. The rates of fuel loads consumption obtained from field experiments vary as a function of the Cerrato main vegetation types^32,61^. We applied the map of vegetation height (h)^66^ to separate areas consisting mostly of grass (h ≈ 0 m), herbaceous plants (0 < h ≤ 1 m), shrubs (1 < h ≤ 2 m) and trees (h > 2 m), so as to assign their respective combustion rates (Table S2).

### Probability of burning

One of the challenges for modeling fire in the Cerrado is the inclusion of the human factor given that most fires are anthropogenic^67^. To do so, we derived the probability of burning (the Bayesian post-probability) given fuel loads as the prior probability and the frequency of burning obtained from MODIS burned area product as the conditional probability^3^. In such a way, we assume that areas that burn more frequently have a higher chance of burning again if enough fuel loads are available^3^.

### Percentage of dry biomass (fuel loads)

We map the percentage of dry biomass as a proxy for fuel loads^3^. In the Cerrado, its open vegetation formations, such as grasslands, shrubs and bushlands, allow the percentage of dry biomass to be gauged using remote sensing^68^. To map the dry biomass percentage, we used the method by Oliveira et al.^3^, which is an adaptation from Franke et al.’s^68^ to generate a single map of continuous values, instead of a RGB composite. This method applies three spectral bands (Red, 640–670 nm; Near Infrared VNIR, 850–880 nm and Short-Wave Infrared SWIR, 1570–1650 nm) to produce a map of spectral mixtures between green and dry vegetation and soil, which is updated daily for MODIS and between 3 and 5 days for Sentinel-2.

### Vegetation moisture

Vegetation moisture is a key factor for estimating the vegetation flammability and in the case of a fire the chances for its extinction. As there is a large variation over time, we inferred the vegetation moisture using a remote sensed index. The method applies the Normalized Difference Water Index (NDWI)—a combination of near-infrared (0.75–1.4 μm) and shortwave infrared (1.4–3.0 μm) bands—to estimate the water content in leaves^54^, thereby allowing the mapping of vegetation moisture at a high spatial resolution (20 m for Sentinel-2) and high frequency as well, i.e., 3–5 days for Sentinel-2 and daily for MODIS (Fig. 3).

## Simulation of fire propagation

The simulation of fire propagation is based on cellular automata (CA) (Table S3 and Fig. S1). The CA simultaneously analyzes the chances of all map cells to catch fire, or to go out, if they are on fire. In addition, the CA calculates the fire intensity (joules) and speed (ms^−1^). The CA consists of five interacting modules (Fig. 3): (1) fire spread dynamics; (2) consumption of fuel loads; (3) fire front speed; (4) fire intensity; and (5) vegetation moisture dynamics (Fig. 3). The result of each module determines the results of all of them in the next time-step. The model equations and constants that govern the behavior of fire in relation to wind, elevation and fuel loads are developed from physical experiments and empirical studies^32,33,35,44,61^.

### Cell flammability

**Figure 4:**
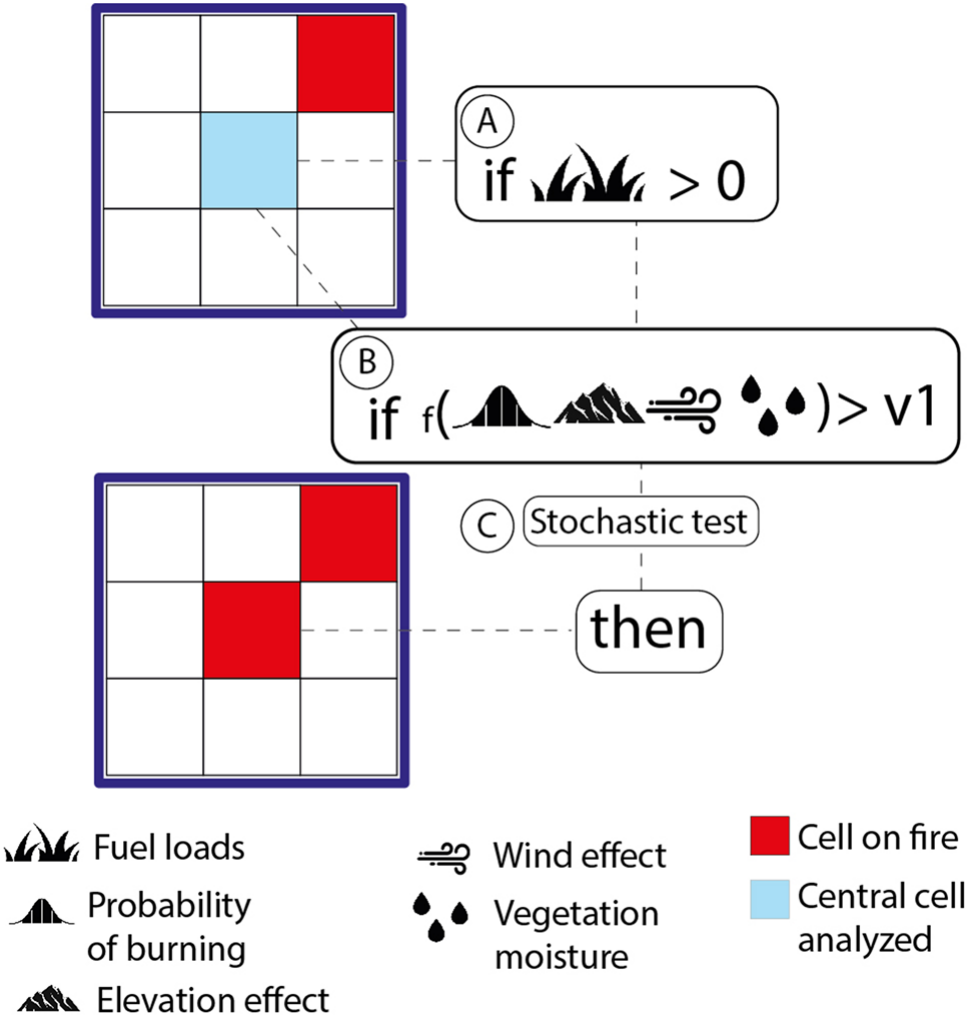
The flammability module. (**A**) test for the presence of fuel loads; (**B**) flammability test combining the probability of burning, topographic and wind effects and vegetation moisture; (**C**) stochastic test to produce radial fire fronts.

**Figure 5:**
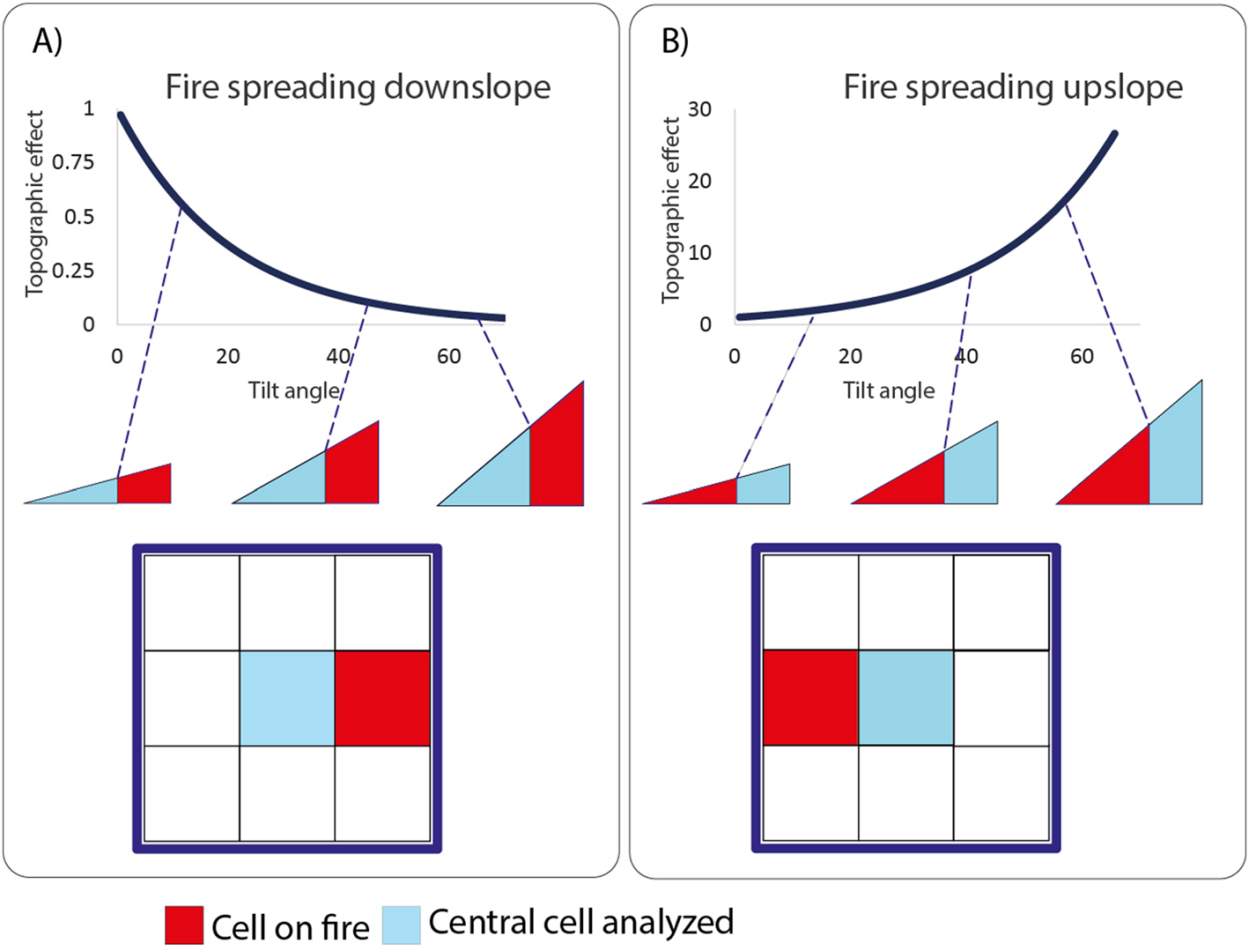
Down slope (**A**) and upslope influence on fire (**B**). The curves indicate the elevation effect (y axis) in relation to slope angles (∅) between central cell and its neighbor on fire (Eq. 1).

In a given step of the CA, for each map cell, the model performs five successive tests to determine whether a cell ignites or not. (1) First, it checks whether there is at least one neighboring cell (Moore-8 neighborhood) on fire or holding a hot pixel (Fig. 4). (2) if true, the central cell may ignite if its fuel loads are greater than zero (Fig. 4A). (3) Next, the model calculates the combined flammability from the probability of burning (Fig. 4B); elevation *fT*^36^ (Fig. 5 and Eq. 1), wind *fW* (Fig. 5 and Eq. 2), vegetation moisture *fM*^69^ and the number of neighboring cells on fire (Eq. 3). 4) If the resulting value is higher than the minimum threshold (*v1,* Fig. 4B) obtained from historical records of burned areas (2001–2020, MODIS product MCD64A1), the cell may catch fire. 5) Finally, the model draws a random number between 0 and 1. A value < 0.42 then triggers the fire (Fig. 4C). This threshold was estimated interactively until the cellular automata could replicate a radial fire front under constant fuel loads, no wind and on a flat surface (Fig. S1).

**Figure 6:**
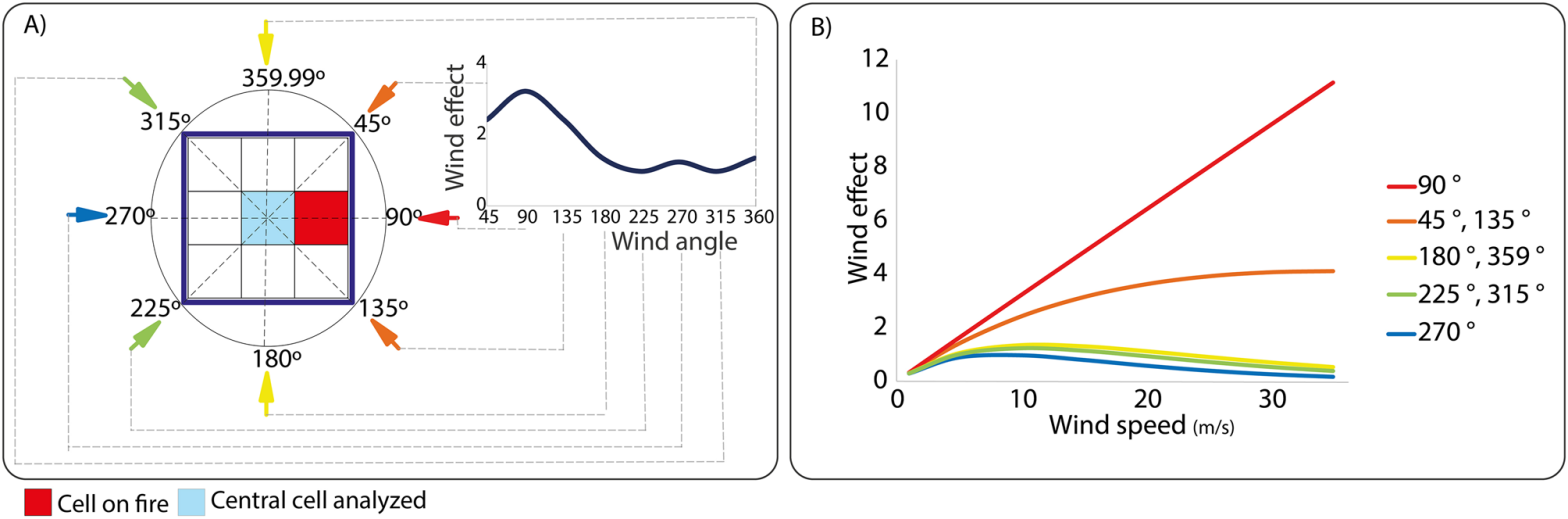
Effects of wind direction (**A**) and wind speed (**B**). In (**A**) the wind effect is a function of the wind angle in relation to the fire direction towards the central cell at a constant wind speed of 10 m/s. The wind effect due to wind speed at different direction angles is shown in (**B**). Red and orange colors indicate the wind directions that favor the propagation of fire towards the central cell (Eq. 2).

The flammability effect due to elevation takes into account whether the neighboring cells on fire are below or above the central cell and the slope gradient between them (Fig. 5, Eq. 1).

1 $$fe=\sum_{i=1}^{8}({e}^{\alpha {\varnothing }_{i}{A}_{\Delta }}*{f}_{i})$$

where *fe* is the flammability effect as a function of the number of neighbors* i* on fire given their elevation in relation to central cell, ∅ is the angular slope, *A*_*Δ*_ is positive when the neighbor *i* is below the central cell and negative when above, *α* is an empirical angular constant and *f* is a binary value, assuming 1 for neighbor* i* on fire^36^. The flammability factor due to wind takes into account the direction and speed of wind coming from neighboring cells on fire. It is positive when the wind blows to the central cell and otherwise negative (Fig. 6).

2 $$fW=\sum_{n=1}^{8}\left({cw}_{1}*{e}^{\left({cw}_{2}*{S}_{i}*(\mathrm{cos}({D}_{i}\right)-1)}\right)*{S}_{i}^{cw3}*{f}_{i})$$

where *fW* is the flammability effect as a function of wind speed and direction from the neighbors *i*, *cw*_*1*_, *cw*_*2*_ an *cw*_*3*_ are empirical constants, *S*_*i*_ is the wind speed in m/s and *D*_*i*_ the angle formed between the wind direction (in degrees) and neighbor *i* relative to the central cell and *f*_*i*_ is a binary value for the presence of fire in the neighbor *i*^69^ (Eq. 2).*fM* is the flammability effect as a function of the vegetation moisture (Eq. 3), where *b*_*1*_ is a constant and *M is* the NDWI index from 0 to 1^69^.

3 $$fM={e}^{(-b1*M)}$$

Finally, *fF* is the combined flammability factor (Eq. 4), given $p\left(B|D\right)$ the probability of burning; *fe,* the elevation effect; *fW,* the wind effect, and *fM,* the flammability from vegetation moisture.

4 $$fF=p\left(B|D\right)*fe*fW* fM$$

### Fire extinction

**Figure 7:**
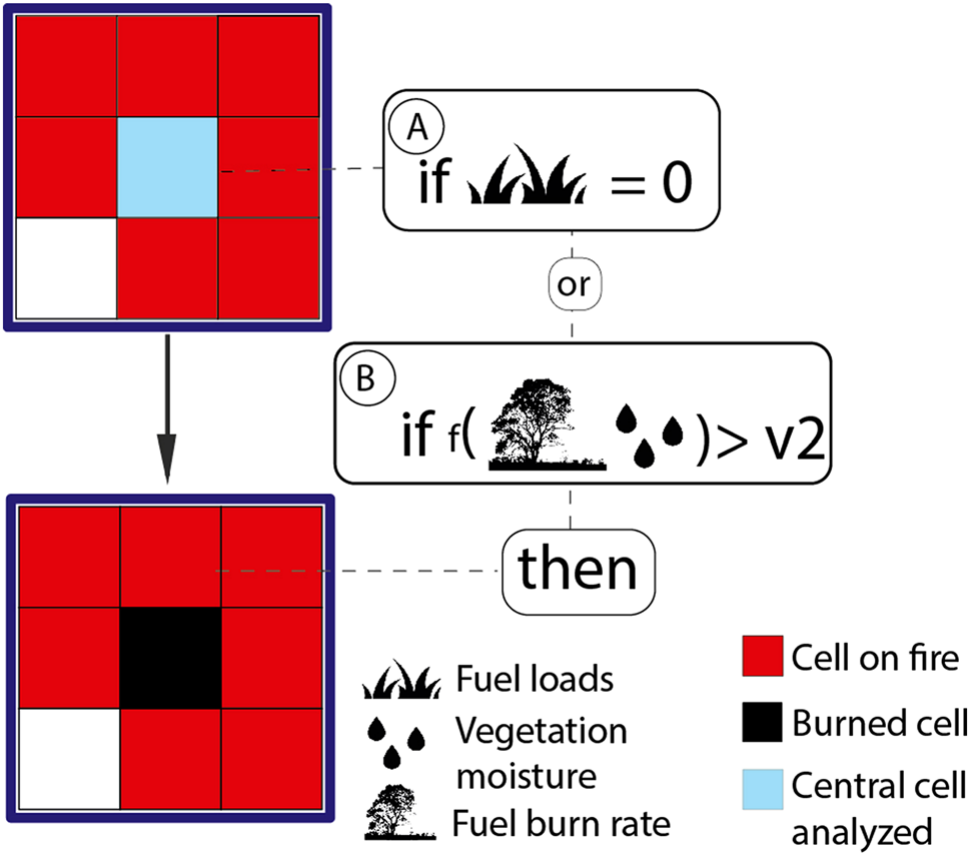
The fire extinction module. The algorithm tests whether the central cell on fire still holds fuel loads (**A**), or if the probability of fire extinction (**B**) given $pE$ (Eq. 5) is greater than *v2*.

For a cell on fire, the fire will go out when there are no fuel loads left. If there are still fuel loads, the model calculates the chance of fire extinction (adapted from Alexandridis et al.^69^) (Fig. 7, Eq. 5) upon the rate of fuel consumption per vegetation type and moisture. If the vegetation moisture is lower than *v2* (Fig. 7), the fire goes out. The value of *v2* is the highest vegetation moisture observed within historical burned areas (MODIS product MCD64A1 from 2001 to 2020).

5 $$pE=V*{1/e}^{{(-b1*M)}^{b2}}$$

where *pE* is the probability of fire extinction given the fuel consumption rate by vegetation type *V, b*_*1*_*, b*_*2*_ are empirical constants^69^ (Table S2), and *M* the vegetation moisture.

### Fuel loads dynamics

For a cell on fire, its fuel loads reduce according to Eq. (6)

6 $${B}_{t}={B}_{t-1}-({B}_{t-1}*C*I)$$

where *B* is the amount of fuel loads at time *t* and *t*_*-1*_, *C* the consumption rate per vegetation type and *I* a flammability factor as follows:

7 $${I=1/(e}^{{\left(-b1*M\right)}^{b2}})$$

*M* is the vegetation moisture, and *b1* and *b2* empirical constants^36^ (Table S2).

### Fire spread speed

The model uses the ratio between the distance propagated by a fire front and its duration to quantify the speed of propagation (m/s). For doing so, the model first identifies individual patches of burned areas, hence the corresponding distance of propagation. Since in the CA, a cell size is fixed, the only variable that needs to be estimated is the model time-step. We used data from controlled experiments that measured fire speed^58^ to estimate the CA time-step. For 20-m spatial resolution, the time step is equivalent to 38.61 s, while for 500 m spatial resolution the time-step is 965.25 s.

### Fire intensity

To estimate the fire intensity, we applied Eq. (8) from Byram^55^, where $In$ is the fire intensity in KJm^−1^ s^−1^, *C is* rate of fuel loads consumption *and D* is the amount of fuel loads consumed in each time-step (Eq. 6) and *S* is the speed of fire propagation.

8 $$In=C*D*S$$

### Vegetation moisture dynamics

Vegetation moisture fluctuates along the day, consequently there is a need to determine the vegetation moisture specifically for the hour of burning^70^. To this end, we modeled the hourly vegetation moisture (*M*_*t*_) by fitting a polynomial curve (Eq. 9) to a 10-years series of minute time-step observations from a set of met-stations located in the Serra do Cipó National Park (Fig. S2). The fit is weighted by the number of neighbors on fire and the fuel consumption rate per vegetation type^71^ (Eq. 9).

9 $${M}_{t}=\left(\left(-4\mathrm{e}-20* {t}^{4}\right)+\left(1\mathrm{e}-14* {t}^{3}\right)-\left(8\mathrm{e}-10* {t}^{2}\right)+\left(2\mathrm{e}-05*t + 0.9734 \right)*M\right)+M))*\frac{N}{100}*C$$

where *Mt* is the moisture at hour *t*, *M* is the daily vegetation moisture, *N* the number of neighbors on fire, *C* the fuel consumption rate and *e* the Euler's number.

## Monte Carlo simulations

The fire spread model is strongly influenced by the predictor variables, most of which come from daily monitoring systems. These variables vary along the day, hence may not represent the environmental conditions on the time a fire is being simulated. For example, wind as a strong determinant of fire behavior may undergo sudden changes in short time intervals and at a local scale, which are seldom detected by the available meteorological systems. An alternative to include these variations in fire predictions is the use of Monte Carlo simulations. By randomly varying the predictor variables (ignition sources, wind, vegetation moisture, and fuel-loads) within a given interval, one can simulate a range of fire propagation scenarios for some specific geographic region. For doing this, we developed offline simulations for each of the nine CUs by changing the predictor variables that are amenable to hourly variation (wind, fuel loads, and vegetation moisture) along with ignition sources, which in this case are allocated randomly. Values for these variables are drawn from a normal distribution centered on the average of observed values obtained from historical records of large fire events, including the spatial distribution of hot pixels. The Monte Carlo simulations have the advantage of generating a panorama of areas that most likely burn within a geographic region (Fig. S3). However, as a disadvantage, there is a high computational cost. For each CU, the 20-m resolution models took 24 h, on average, to run 1000 rounds. The resulting probability maps are used to indicate the most prone areas to fires as a means to plan firebreaks aimed at protecting sensitive woodlands under an Integrated Fire Management Program of ICMBio.

## Local interactive version

For simulating fire spread from locations determined by the user in order to test the viability for prescribed burning, we provide a local interactive version of FISC-Cerrado. This version, also developed using Dinamica EGO^47^, comes with a wizard-interface for easy and customized set up (Fig. S4). The user needs only to specify the geographic coordinates for ignition points and choose the dates and surrounding area for simulating a fire. The user-interface is simple and as such can be used by anyone without any specialization or previous training. Although the model runs on a local computer, the input data (wind, vegetation moisture, probability of burning and fuel loads) are constantly and automatically updated from the FIP-Cerrado online platform, hence eliminating the local processing of input data. For future date of simulation, the local model adjusts the input data (fuel loads and vegetation moisture) up to 20 days after the last satellite image acquisition by using polynomial curves fit to historical data (2015–2020) (Eq. S1).

## Model validation

**Figure 8:**
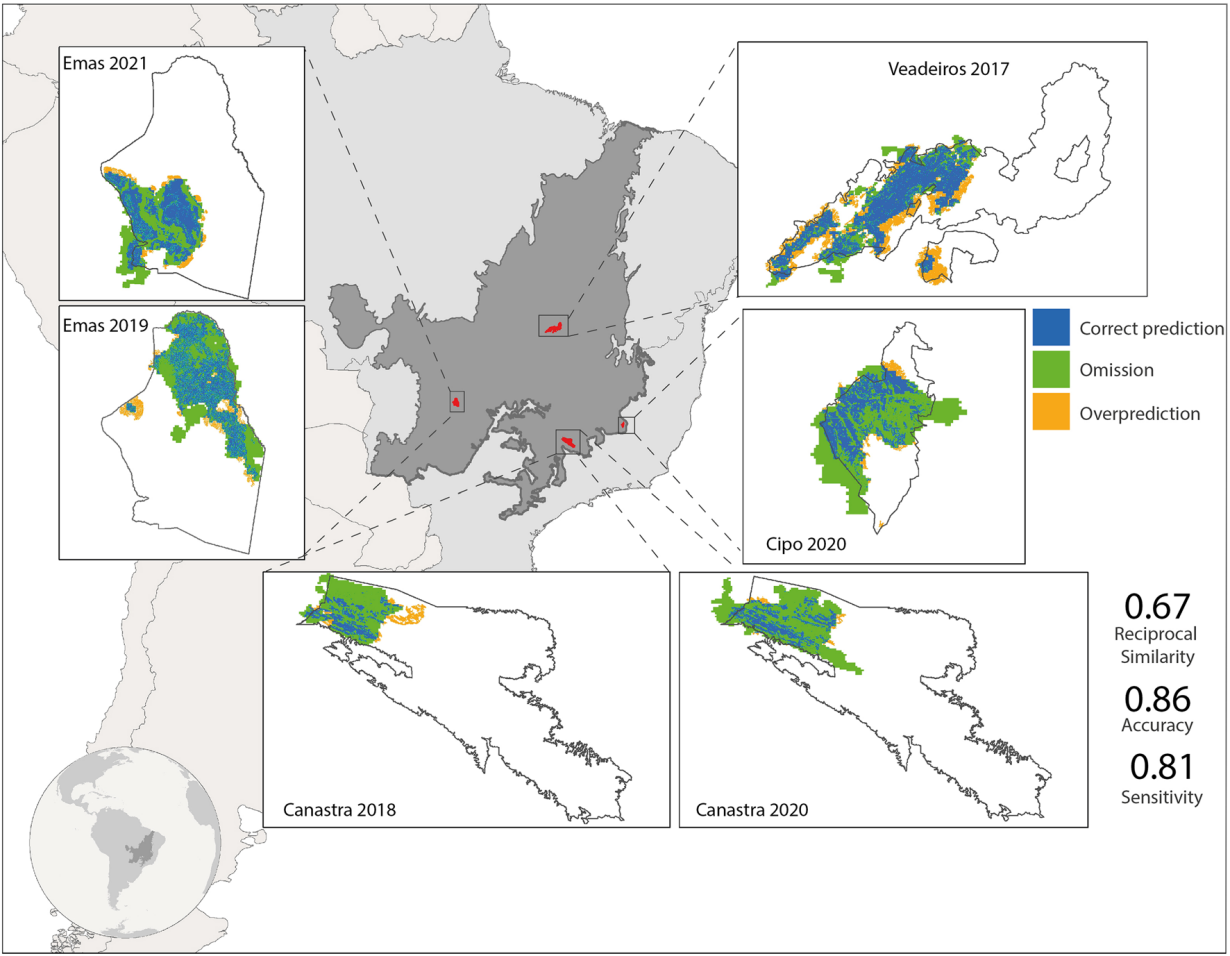
Validation of FISC-Cerrado. Simulated fire scars (20 m) laid over burned areas from MODIS^64^. Average validation values depicted on the right bottom. Map created in ArcGIS 10.1 (http://www.esri.com).

To test the predictive power of our model, we selected six wildfire events that occurred in the Cerrado between 2006 and 2020 that were not tamed by fire brigades in order to avoid inflating overprediction errors (Table S4). Those selected wildfires located in the CUs of Serra Canastra, Emas, and Chapada dos Veadeiros (Figs. 8 and S5). As ground truth, we used the respective final burned areas from MODIS for validating our predictions as well as the Monte Carlo simulations. As the validation metrics, we applied a map comparison metric—i.e., the Reciprocal Similarity Comparison with exponential decay^47^—, and the accuracy^72^ and sensitivity^72^ tests, which assess the overall hit and the presence hit rate, respectively. The methods only consider the area formed by union of the extents of both observed and simulated fires, hence eliminating the unchanged areas—a mandatory procedure for assessing spatial matching. The validation was performed for simulations with two spatial resolutions, 500 and 20 m. To validate the Monte Carlo simulations, we calculated the Area Under the Curve (AUC)^72^, comparing the probability values with the maps of the same selected fire events.

As a result, the fire spread model showed a high predictive capacity for the 20-m resolution (reciprocal similarity = 0.67, accuracy = 0.86 and sensitivity = 0.81) and the 500-m resolution version (reciprocal similarity = 0.65, accuracy = 0.89 and sensitivity = 0.71), hence with little difference in the predictive capacity for both resolutions. These values are higher than those obtained from a null model (i.e., with constant fuel loads and no wind, Fig. S6). However, the 500-m model yielded lower precision values due to overprediction (Fig. 8). The fire spread probability from the Monte Carlo simulations showed a high predictive capacity (average AUC = 0.92). The highest AUC was 0.98 for the Canastra Park, 2018 fire, and the lowest of 0.87 for Emas Park, 2019 fire.

## Utilization of FISC-Cerrado and future prospects

Our fire spread model holds a good predictive ability for large fires both in the Cerrado as a whole and for the CUs’ regions as well. Results from validation also point out that even without high precision wind data, the model can still make good predictions under different environmental conditions. Hence, FISC-Cerrado not only puts together the science of fire into a single modeling framework, it also validates many research findings^35,36,55,58,61,69^, furthering new studies to overcome the main limitations of today.

In fact, none of initiatives to date, whether global^2^ or regional^2,30,31,73^, provide a whole set of solutions as those available on the FISC-Cerrado platform (both online and local versions), especially regarding the near real-time prediction of fire spread in a completely automated way. And even the most recent initiatives aimed at modeling fire spread over vast areas^74^ do not come close to the spatial resolution (20 m) of our thrice-a-day simulations for the nine CUs' regions that encompass about 6.1 Mha, not to mention the 213 Mha of the entire Cerrado. These simulations process 36 GB of data per run, on average, which takes from 30 min. to 2 h (depending on the number of ignition sources) on a server with 80 cores of 2.77 GHz (of which the model utilizes simultaneously 40) and 768 GB RAM. This efficiency in data processing is only possible thanks to Dinamica-EGO 7*. Its architecture enables massive data processing by running on parallel multiple tasks and regions, such as the individual CUs, while simultaneously preparing the outputs for publication. Moreover, the integration of EGO operators with Python and R codes together with the Dinamica EGO visual programming interface allows the complete automatization of tasks, including data downloading, processing, and publishing. In addition, the Mappia map server enables the quick display of the massive dataset output from the model on an online dashboard containing interactive maps and charts. And all of this is freely available. In order to disseminate our expertise, all model components and submodels developed specifically for our project can be found at the EGO online library of operators. As such, they can be downloaded, promptly used or opened as an individualized model to be adapted and reused for new developments.

Unlike the requirements to operate fire models, such as the American FARSITE^29^ and the Canadian Prometheus^30^, the user-friendly interface of FISC-Cerrado Platform, alongside the automatization of the entire chain of tasks, allows its use by practitioners who do not have technical skills, such as GIS knowledge. The platform has been also developed tailored to end-users’ needs who requested the addition of a series of ancillary information to help their daily field operation, such as the Planet high-spatial resolution images. Finally, to support the utilization of the Platform, we have a carried out a comprehensive training program that enabled its utilization by ICMbio technicians for planning integrated fire managing and guiding firefighting operations in the monitored CUs (https://csr.ufmg.br/fipcerrado/en/). Thus, the FISC-Cerrado platform introduces an advanced territorial intelligence tool for the open and user-friendly access by a broad community of practitioners from operational fire brigades to policy makers. In face of ever-more destructive wildfires, such a tool is becoming increasingly needed to optimize prevention and firefighting campaigns both in terms of their costs and time-response. Increasing our effectiveness in taming wildfires in vast regions is vital for biomes like Cerrado, which still shelters large tracts of native vegetation.

For doing so, there is a need for continued public investments in wildfire mitigation. Despite experiencing mounting wildfires, Brazil invests but ≈US$ 3 ha^−1^ year^−1^, on average, in fire prevention and mitigation in the national CUs of the Cerrado and Amazon^7^. Although a paltry figure when compared with those invested in the Global North^7^, it has made a difference in reducing fire occurrence in those areas. The Forest Investment Program of the Climate Investment Fund through the World Bank invested about US$ 2.5 M over 5 years in the development of the FISC-Cerrado platform. According to the Brazilian Association for Development, this project paid back in benefits to the society USD 5.89 for each dollar invested^75^. However, the lack of continued investments puts in risk the future of FISC-Cerrado that has proven to be an essential tool for helping mitigate wildfires. This platform also needs to be expanded to the Amazon, where research on fire modeling has already made some headway^56,57^, and Pantanal, which together with the Cerrado are the biomes where high-impact fires occur^1^. In this respect, the use of physical principles of fire behavior as the basis for FISC-Cerrado makes it easy to adapt its architecture to other Brazilian biomes and regions of the world.

Well-funded research on the ecology of fire in the various terrestrial biomes will be central to underpin modeling development. There is also a need for higher spatial and temporal resolution wind data from an enhanced network of MET stations. In parallel, progress in computer performance to handle high spatial-resolution simulations for large areas, e.g., 20 m, will be mandatory. Future enhancements could include, for example, the logistics for mobilizing fire brigades, especially in remote areas of the Amazon, parallel processing of individual fire propagation fronts, incorporation of burned areas from high temporal frequency sensors like VIRS, and the prompt and easy communication of alerts via social networking channels. And, as importantly, this must be attained at a moderate computer cost by continuing developing Dinamica EGO freeware that is tailored to take advantage of gaming computers, which in a distributed computing system can perform on a par with high-end computers and clouds. Under the current climate crisis, those innovative tools when used in concert with smart field operations not only will help mitigate the socioeconomic and ecological burden of wildfires, it will benefit the society as whole.

## Supplementary Information

Supplementary Information.

## Data Availability

FISC components are available as submodels on Dinamica EGO online store (www.dinamicaego.com), Mappia elements and code are available on https://mappia.earth and data used in the FISC-model at FISC-Cerrado online platform.
