# Supplementary material for: A near real-time web-system for predicting fire spread across the Cerrado biome
Source: Sci Rep. 2023 Mar 24;13:4829. doi: 10.1038/s41598-023-30560-9 (PMC10039015; doi:10.1038/s41598-023-30560-9)
Supplement: Supplementary file 1 — Supplementary Information. [file 41598_2023_30560_MOESM1_ESM.docx]

Supplementary Materials for

**A near real-time web-system for predicting fire spread across the Cerrado Biome**

Ubirajara Oliveira^*^, Britaldo Soares Filho, Hermann Rodrigues, Danilo Figueira, Leticia Gomes, William Leles, Christian Berlinck, Fabiano Morelli, Mercedes Bustamante, Jean Ometto, Heloísa Miranda

*Corresponding author: ubiologia@yahoo.com.br

**This PDF file includes:**

Figs. S1 to S6

Tables S1 to S4

Equation S1


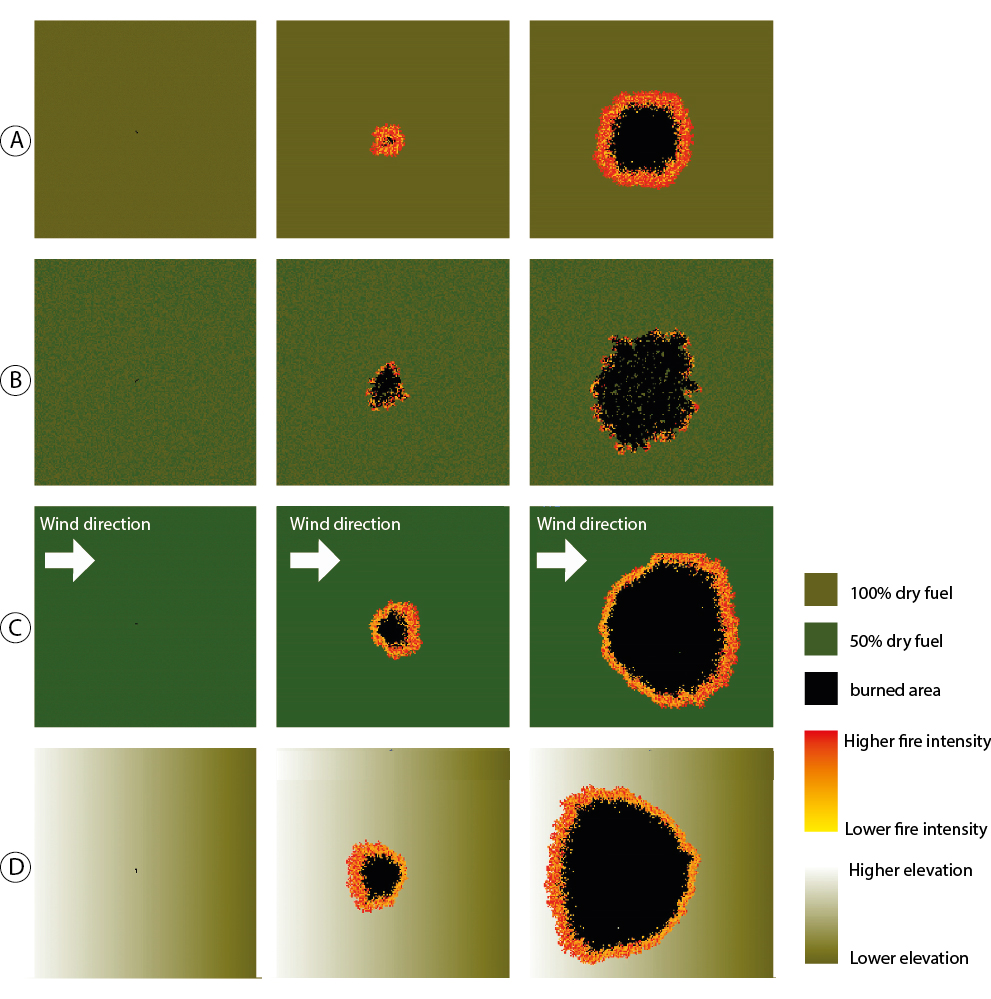


Fig. S1. Fire spread tests. A) control conditions, without wind, slope =0 and constant moisture; B) with random moisture, without wind and slope = 0; C) with wind; and D) with slope > 0.

Fig. S2. Blue points, average moisture measured at six meteorological stations in *Serra do Cipó* (2011 - 2018). Red points depict the polynomial model.


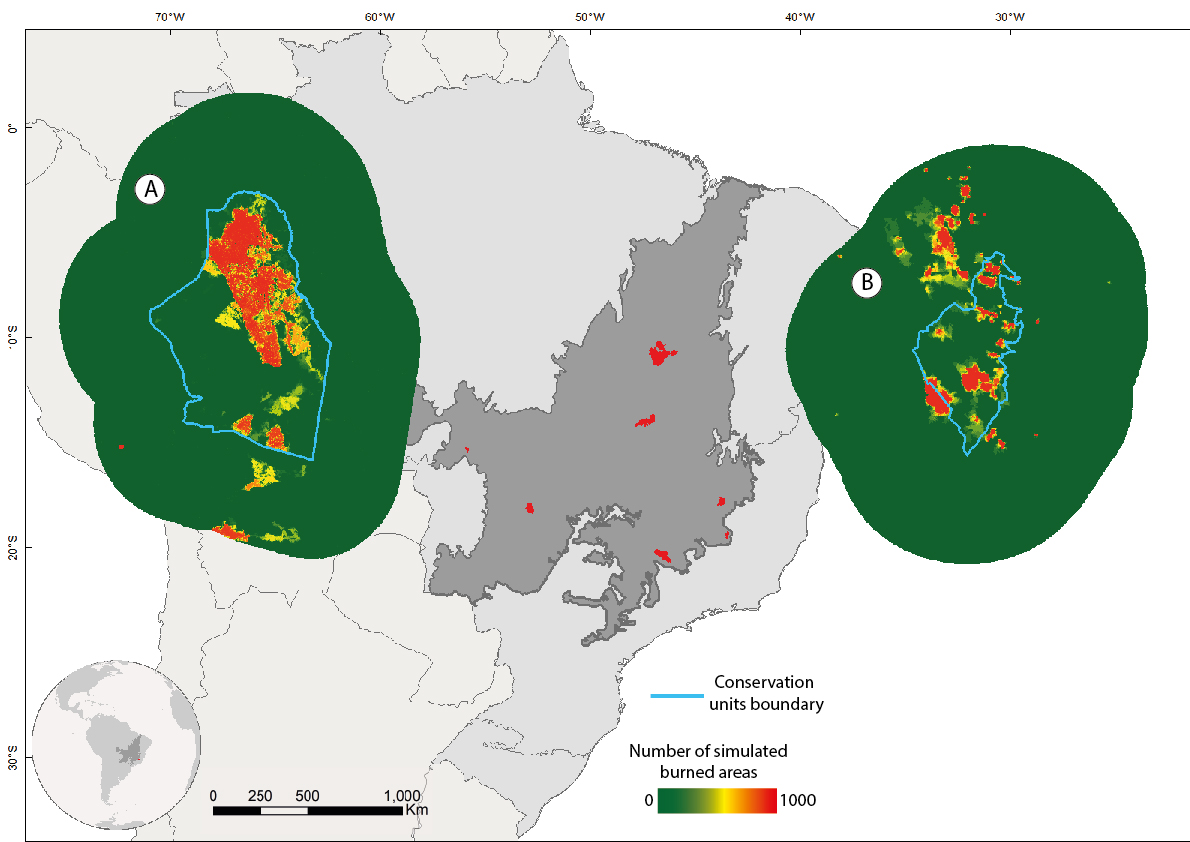


Fig. S3. Probability maps from Monte Carlo simulations for A) Emas National Park and for B) Serra do Cipó National Park in B). The areas surrounding the conservation units are also included in the Monte Carlo simulations. The bar color indicates the propensity to fire (from 0 to 100). Map created in ArcGIS 10.1 (http://www.esri.com).


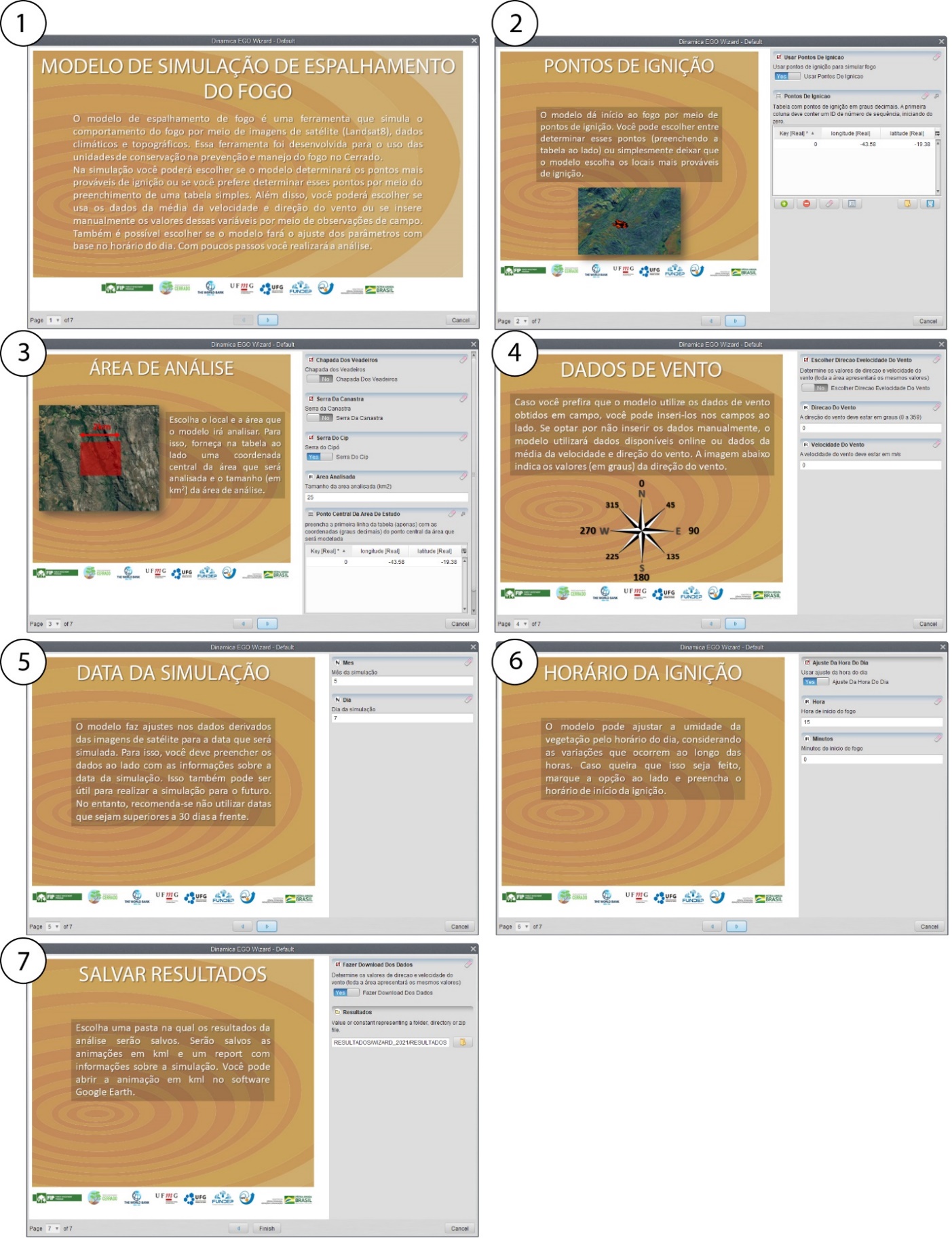


**Fig. S4. Wizard-interface for easy and customized set up.** 1: Model presentation, 2: Ignition source coordinates, 3: CU selection and area boundary, 4: Wind direction and speed, 5: Simulation date; 6: simulation hour, 7: folder selection for saving results.

| 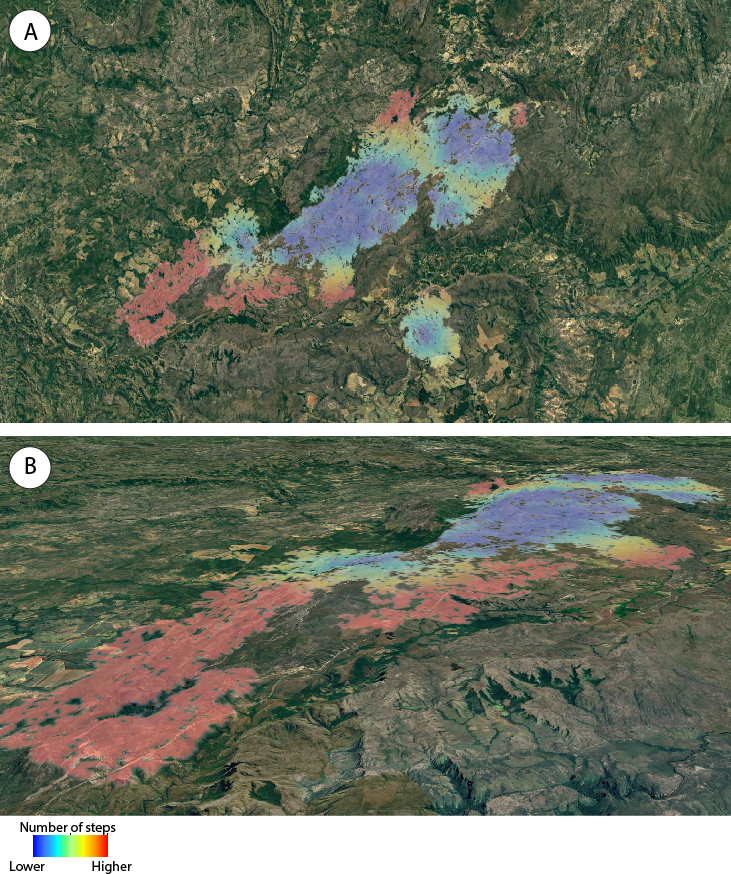 |
| --- |
| **Fig. S5. Simulated fire in the Chapada dos Veadeiros Park.** A) nadir view and B) 3 D view over Google Earth. Color bar indicates the number of time-steps a fire has propagated from its ignition source. Map created in Google Earth (https://earth.google.com). |

|  |
| --- |
|  |
| 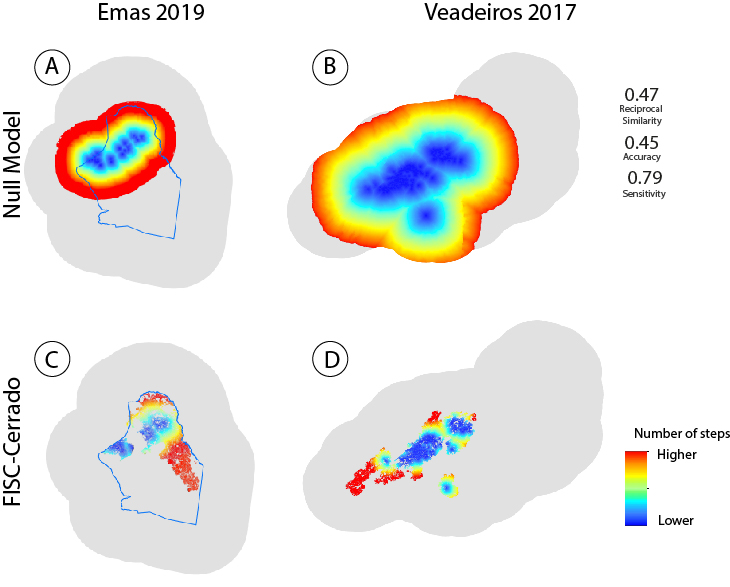 |
| Fig. S6. Null Model and FISC-Cerrado simulations. A and B: Null model simulations for Emas and Chapada dos Veadeiros; C and D: FISC-Cerrado Simulation for the same parks. Average validation values for null models depicted on the right top. Color bar indicates the number of time-steps a fire has propagated from its ignition source. |

Table S1. Satellites used for mapping hot pixels.

| Instrument | Spatial resolution | Temporal frequency |
| --- | --- | --- |
| Terra-MODIS | 250m | 1 day |
| Aqua-MODIS | 250m | 1 day |
| GOES16 | 1km | 30 minutes |
| NOAA-18 | 1.1km | 2 hours |
| NOAA-19 | 1km | 2 hours |
| MSG-03 | 7km | 15 minutes |
| METOP-B | 1km | 1 day |
| METOP-C | 1km | 1 day |
| NPP-375 | 750m | 1day |

Table S2. Values for empirical constants. *α* is an empirical angular constant, *cw_1_*, *cw_2_* and *cw_3_* are empirical constants for the wind equations, and *b_1_ and b_2_* empirical constants for the vegetation moisture equation.

| Empirical Constant | Value | | Equation | Reference |
| --- | --- | --- | --- | --- |
| *α* | | 0.05 | 1 | Almeida, R. M. & Macau, E. E. N. Stochastic cellular automata model for wildland fire spread dynamics. J. Phys. Conf. Ser. 285, 12038 (2011). |
| *cw_1_* | | 0.33 | 2 | Alexandridis, A., Vakalis, D., Siettos, C. I. & Bafas, G. V. A cellular automata model for forest fire spread prediction: The case of the wildfire that swept through Spetses Island in 1990. Appl. Math. Comput. 204, 191–201 (2008). |
| *cw_2_* | | 0.99 | 2 | Alexandridis, A., Vakalis, D., Siettos, C. I. & Bafas, G. V. A cellular automata model for forest fire spread prediction: The case of the wildfire that swept through Spetses Island in 1990. Appl. Math. Comput. 204, 191–201 (2008). |
| *cw_3_* | | 0.06 | 2 | Alexandridis, A., Vakalis, D., Siettos, C. I. & Bafas, G. V. A cellular automata model for forest fire spread prediction: The case of the wildfire that swept through Spetses Island in 1990. Appl. Math. Comput. 204, 191–201 (2008). |
| *b_1_* | | 0.0001 | 3 | Alexandridis, A., Vakalis, D., Siettos, C. I. & Bafas, G. V. A cellular automata model for forest fire spread prediction: The case of the wildfire that swept through Spetses Island in 1990. Appl. Math. Comput. 204, 191–201 (2008). |
| *b_2_* | | 0.0005 | 5 | Alexandridis, A., Vakalis, D., Siettos, C. I. & Bafas, G. V. A cellular automata model for forest fire spread prediction: The case of the wildfire that swept through Spetses Island in 1990. Appl. Math. Comput. 204, 191–201 (2008). |
| *v1* | | 0.48 |  |  |
| *v2* | | 0.59 |  |  |

**Table S3. Simulation videos**

| Simulation | link |
| --- | --- |
| PARNA da Serra do Cipó | <https://www.youtube.com/watch?v=1G1khvFIKrU&t=21s> |
| PARNA da Serra da Canastra | <https://www.youtube.com/watch?v=Z_O0-QuBw5c> |
| PARNA da Chapada dos Veadeiros | <https://www.youtube.com/watch?v=9avKyb-2oIU> |

The colors follow the same color bar of Figure S1. FISC website allows downloading the simulation results in KMZ format that enables this type of animation on Google Earth.

Table S4. News about wildfire events used in FISC-Cerrado validation.

| Fire Event | date | fonte |
| --- | --- | --- |
| Canastra_2018 | 21/08/2018 | <https://g1.globo.com/mg/centro-oeste/noticia/2018/08/23/incendio-no-parque-nacional-da-serra-da-canastra-chega-ao-3-dia-brigadistas-e-bombeiros-seguem-em-meio-a-mata.ghtml> |
| Canastra_2020 | 20/08/2020 | <https://g1.globo.com/mg/centro-oeste/noticia/2020/08/20/chega-ao-fim-o-incendio-na-serra-da-canastra-apos-6-dias-de-combate-e-quase-24-mil-hectares-atingidos.ghtml> |
| Emas_2019 | 03/09/2019 | <https://www.correiobraziliense.com.br/app/noticia/cidades/2019/09/03/interna_cidadesdf,780602/incendio-no-parque-nacional-das-emas-consome-6-mil-hectares-do-parque.shtml> |
| Emas_2021 | 13/07/2021 | <https://g1.globo.com/go/goias/noticia/2021/07/13/incendio-no-parque-nacional-das-emas-e-controlado-apos-atingir-mais-de-20-mil-hectares-diz-diretor.ghtml> |
| Veadeiros_2017 | 18/10/2017 | <https://www.jornaldocomercio.com/_conteudo/2017/10/geral/590792-incendio-no-parque-da-chapada-dos-veadeiros-esta-controlado-mas-surge-novo-foco.html> |
| Cipo_2020 | 06/10/2020 | <https://www.em.com.br/app/noticia/gerais/2020/10/06/interna_gerais,1192266/depois-de-10-dias-incendio-e-controlado-na-serra-do-cipo.shtml> |

**Equation S1**

$pFl=\left( 2e-13 * d^{5}+ 3e-11 * d^{4}- 9e-08 * d^{3}+ 3e-05 * d^{2}- 0.0023 * d+ 0.288 \right)-\left( oFl \right)*aFl$ Eq. (S1)

Where $pFl$ is the estimated fuel loads at day *d*, *oFl* is the observed fuel loads mean, *cFl* is the average of fuel loads*,* and *e* the Euler's number.
